# Linear asymptomatic pneumatosis as an unexpected finding of computed tomography colonography: a case report

**DOI:** 10.1186/1752-1947-7-205

**Published:** 2013-08-14

**Authors:** Nicola Flor, Paola Pricolo, Antonio Rovere, Miriam Mezzanzanica, Mauro Peri, Gianpaolo Cornalba

**Affiliations:** 1 Unità Operativa Radiologia Diagnostica e Interventistica, Azienda Ospedaliera San Paolo, via A di Rudinì 8, 20142 Milano, Italy; 2 Dipartimento di Scienze della Salute, Università degli Studi di Milano, Milano, Italy

## Abstract

**Introduction:**

We discuss asymptomatic colonic pneumatosis, an unexpected finding of computed tomography colonography that we must see as distinct from perforation. Among the papers detailing complications with computed tomography colonography, we found only one report focusing on linear pneumatosis.

**Case presentation:**

We report the case of a 75-year-old Caucasian woman who had a high level of carcinoembryonic antigen, and who underwent computed tomography colonography. Our patient accidentally fell from a chair in the radiology department just before the examination, experiencing a right hip trauma. The examination was negative for colonic lesions but revealed the presence of some air bubbles in her right colon. Our patient remained asymptomatic throughout the procedure and afterwards; no intervention or treatment was necessary.

**Conclusion:**

Radiologists should consider colonic linear pneumatosis among the potential complications of computed tomography colonography, even if it is a rare event, to avoid unnecessary therapy and anxiety for the patient.

## Introduction

Computed tomography colonography (CTC) is a valid alternative to optical colonoscopy (OC), sharing its high rates of accuracy in diagnosis of polyps and colon cancers [1-5]. Part of the success of the method is due to its low invasiveness, which is clearly lower than OC [6-8]. Complications are quite rare, and amongst these the most feared is intestinal perforation. The report we present here concentrates on an unexpected CTC imaging finding, which must be seen as distinct from perforation: intestinal pneumatosis.

## Case presentation

We present the case of a 75-year-old Caucasian woman with an increase of carcinoembryonic antigen three months after an incomplete OC, who underwent CTC to exclude the presence of colonic lesions. The OC was incomplete and interrupted at her sigmoid colon because of inadequate bowel cleansing. Our patient was being followed-up for lung cancer. She had carried out intestinal preparation in the days preceding the CTC with a mild laxative associated with a low fiber diet. On the morning of the examination, just before taking the oral contrast medium for fecal tagging, our patient accidentally fell from a chair in the radiology department, experiencing a right hip trauma. Because of the persistent pain, she immediately underwent a pelvis X-ray, which excluded fractures.

**Figure 1:**
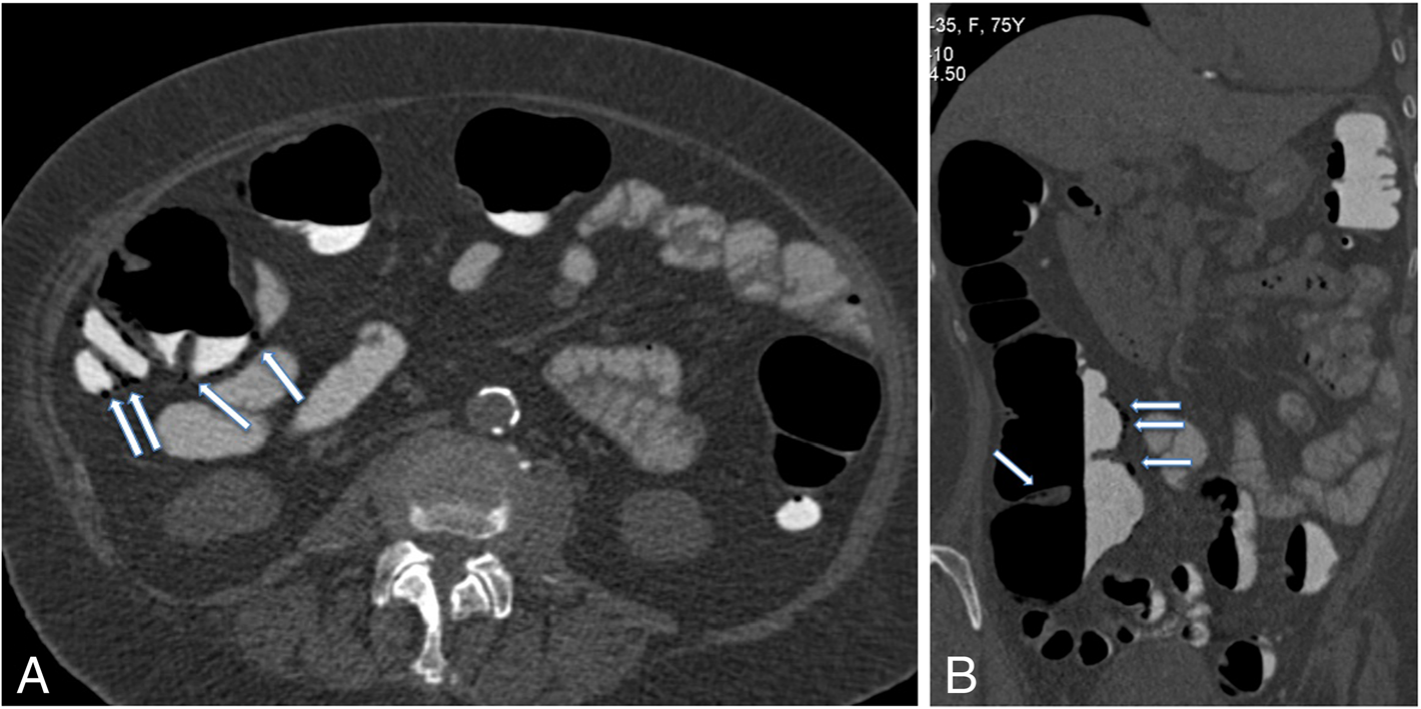
**Two-dimensional computed tomography colonography images. (A)** Supine axial and **(B)** right lateral coronal images show tiny curvilinear pneumatosis (arrows) involving the ascending colon, ileocecal valve and cecum without involvement of the transverse and left colon.

The CTC was performed as usual, using an automated insufflator for colon distension that administered about 2.6L of carbon dioxide, which was well tolerated. We did not use a hypotonic drug. The scans were obtained at end expiration with our patient in the supine position and in the right lateral decubitus position; the prone decubitus was not possible because of the difficulties our patient had in keeping that position. There were no lesions in her colon but numerous tiny air bubbles were present in her right colon (Figure 1), and in particular in her cecum, ileocecal valve and ascending colon, as in cases of intestinal linear pneumatosis, without free intraperitoneal gas. Pneumatosis was already evident at the first scan and no deterioration was shown in the second scan of the lateral decubitus, taken two or three minutes after the first. Our patient was asymptomatic after the examination and went home; she was contacted by telephone that evening and in the following days, never reporting symptoms. She underwent no further medical treatment.

## Discussion

Asymptomatic colonic pneumatosis is a rare potential CTC complication. Among papers discussing CTC complications, there is one report dealing with pneumatosis cystoides coli [9] and only one, by Pickhardt *et al.*[10], focusing on linear pneumatosis. Pickhardt *et al.* described six cases of linear colonic pneumatosis in 5,368 patients who underwent CTC examination for screening purposes, with a prevalence of 0.11%. They reported that pneumatosis was always asymptomatic and correlated with the use of automated insufflators of carbon dioxide. Our case has some analogies with that report [10]. Our patient was also asymptomatic; the pneumatosis was detected at the end of the examination, during the first review of the images, and represented a surprising finding due to the absolute absence of symptoms during the CTC and immediately after the procedure. Moreover, our patient judged the examination as absolutely not painful in her questionnaire after the CTC. This unexpected finding also occurred using a carbon dioxide automated insufflator for colonic distention. We have used this device for 15 months instead of room air insufflation: this is the only case of pneumatosis that occurred out of a total of almost 800 CTC examinations (0.1%) performed with the use of an automated insufflator. In almost 700 examinations performed with room air, a similar event had never occurred.

The cause of this event is unclear. Some authors [11-14] have postulated the role of different factors including intraluminal pressure, mucosal integrity, bacterial flora and intraluminal gas. Pickhardt *et al.* hypothesized a potential role for cathartic bowel preparation [10].

We cannot exclude that linear pneumatosis in our case resulted from the traumatic event, which may have caused an acute increase in intraluminal pressure and, subsequently, a mucosal laceration.

Kelly *et al.*[15] described a case of portal venous air embolism and extensive pneumatosis cystoides intestinalis secondary to blunt abdominal trauma, diagnosed by conventional CT.

These findings are not the sole prerogative of CTC; it is likely that such asymptomatic findings occur more frequently on OC, but are not recognized without CT imaging.

Linear pneumatosis should not be confused with perforation and with so-called primary pneumatosis cystoides coli, which typically manifests itself as a cluster of air-filled cysts usually involving the left colon, and resembling polyposis on endoluminal evaluation.

## Conclusion

Radiologists dealing with CTC have to consider linear colonic pneumatosis as a potential complication, even if it represents a rare event, to avoid unnecessary therapy and anxiety for the patient. We therefore suggest this unexpected imaging finding be included in the patient’s information sheet.

## Consent

Written informed consent was obtained from the patient for publication of this case report and accompanying images. A copy of the written consent in available for review by the Editor-in-Chief of this journal.

## Competing interests

The authors declare that they have no competing interests.

## Authors’ contributions

PP and AR performed the CTC examination and were involved in drafting and revising the manuscript; MM, MP and GC were involved in drafting and revising the manuscript. NF was a major contributor in writing the manuscript. All authors read and approved the final manuscript.
